# Early-Phase Oncology Research Training (EFFORT) program: a Hengrui-USCACA joint scholarship

**DOI:** 10.1186/s40880-015-0035-5

**Published:** 2015-07-08

**Authors:** Li Yan, Li Xu

**Affiliations:** The US Chinese Anti-Cancer Association, Martinez, CA 94553 USA; Beijing Cancer Hospital and Institute, Peking University School of Oncology, Beijing, 100142 P. R. China; Jiangsu Hengrui Medicine Co., Ltd, Shanghai, 200122 P. R. China

Early-Phase Oncology Research Training (EFFORT) program is a joint effort between Jiangsu Hengrui Medicine Co., Ltd, China and the United States–Chinese Anti-Cancer Association (USCACA) to provide academic and financial support to junior clinical research staff including physicians and nurses of early-phase oncology clinical trial centers in China. The program will select 1–2 qualified candidates per year and place them through rotation programs at 2–3 phase 1 cancer centers in the United States (US).

Current US cancer centers engaged in the EFFORT program include Wisconsin University Comprehensive Cancer Center, H. Lee Moffitt Cancer Center & Research Institute, and MD Anderson Cancer Center. The program also includes a rotation at Cancer Therapy Evaluation Program (CTEP) at National Cancer Institute. The duration of rotation is 4–8 weeks at each center. The exchange scholars will go through a structured clinical rotation with curriculum specifically designed for the individual needs and tailored for special features at each cancer center. The rotation at CTEP focuses on the clinical trial design, and the candidate will be part of the multi-disciplinary team to review, discuss, and evaluate study proposals under the guidance of experienced CTEP medical staff. For more information about EFFORT, please see the article by Dr. Jingji Yang detailing the experiences from the pilot EFFORT program [1].

The program is overseen by a Governance Committee that consists of the following members.

- Shu-Kui Qin, Nanjing Bayi Hospital, China; Immediate Past President of Chinese Society Clinical Oncology (CSCO) and the President of CSCO Foundation
- Lin Shen, Beijing Cancer Hospital, China
- Weijing Sun, University of Pittsburg, USA
- Yi-Long Wu, Gaungazhou General Hospital, China; President of CSCO
- Li Xu, Vice President and Global Head of Oncology, Jiangsu Hengrui Medicine Co., Ltd; USCACA
- Jie Wang, Beijing Cancer Hospital, China
- Rui-Hua Xu, Sun Yat-sen University Cancer Center, China
- Li Yan, Beijing Cancer Hospital, China; USCACA
- Cai-Cun Zhou, Shanghai Pulmonary Hospital, Tongji University School of Medicine, China

The responsibilities of the Governance Committee include implementing the EFFORT program in China, e.g., distributing the program information through professional organization (i.e., CSCO), hospitals, and cancer centers; identifying, reviewing, and qualifying candidates.

The EFFORT program is open to all junior clinical research staffs in academic research centers that are accredited for phase 1 studies on oncology. In principle, the expenses of rotation program are shared between the candidates’ home institution in China and USCACA. USCACA is also responsible for designing the rotation program in collaboration with the home institution and the candidate.

The implementation of the EFFORT program is led by Drs. Roger Luo and Pascal (Jiang) Qian of USCACA. To apply, please contact USCACA at luofr@yahoo.com and pascal.qian@novartis.com. Please send your application with a brief introduction of your background and experience in clinical oncology trials, any particular aspects of the early clinical trials you are looking to strengthen, together with curriculum vitae (CV), the letter of support from the head of department and hospital providing the vision of your institution in early phase clinical oncology trials, and the rationales why your participation in the program will benefit your home institution.

USCACA wants to take this opportunity to thank the following individuals in the US for their support to the Hengrui-USCACA Scholarship.

Dr. George Wilding and Ms. Dona Alberti, University of Wisconsin Carbone Cancer Center

Drs. Wei Sheng and Dan Sullivan, Moffitt Cancer Center

Drs. Helen Chen and Pamela Harris, CTEP at National Cancer Institute

Drs. David Hong, Funda Meric-Bernstam, Denise De La Cruz, and Sarina Anne Piha-Paul, MD Anderson Cancer Center

The program would be impossible without the generous grant from Jiangsu Hengrui Medicine Co., Ltd, China. The USCACA want to thank Mr. Piaoyang Sun, Drs. Li Xu, and Lianshan Zhang for their support of the program.
